# Implementing Quality Improvement Initiatives Within Community Psychiatry: Challenges and Strategies

**DOI:** 10.1007/s10597-024-01375-3

**Published:** 2024-11-25

**Authors:** Lucy Ogbu-Nwobodo, Anya Fang, Harminder Gill, Sam Ricardo Saenz, Paul Wallace, Christina Mangurian, Johanna B. Folk

**Affiliations:** 1 Department of Psychiatry and Behavioral Sciences, University of California San Francisco, San Francisco, CA USA; 2 Department of Psychiatry & Behavioral Sciences, Stanford University, Stanford, CA USA; 3 Department of Psychiatry and Human Behavior, Brown University, Providence, RI USA

**Keywords:** Quality improvement, Public psychiatry, Mental health disparities, Health equity

## Abstract

Implementation of quality improvement (QI) initiatives within community mental health settings is crucial to addressing equity-related issues affecting mental health services delivery, including for co-occurring substance use disorders. Given the growing recognition of QI interventions as an effective framework to facilitate structural change within systems of care, it is important to equip mental health providers with the knowledge and ability to execute QI initiatives that are feasible, sustainable, and integrate a health equity lens. To demystify the QI process, we describe the design and methodologies of four fellows’ capstone projects conducted during the 2022–2023 academic year at the University of California, San Francisco (UCSF) Public Psychiatry Fellowship at Zuckerberg San Francisco General Hospital and Trauma Center (ZSFG). By highlighting fellows’ experiences with leading QI initiatives within community mental health settings, we discuss strategies for overcoming implementation barriers including stakeholder engagement and transparency factors, resource and time constraints, unexpected changes in direction, and lack of infrastructure for QI. Lastly, we reflect on best practices and sustainability considerations for leading QI initiatives in partnership with academic centers, departments of public health, and community mental health clinics.

## Introduction

Public and community psychiatry practitioners serve the most marginalized psychiatric populations in safety net settings (Sowers et al., 2023). These providers must commonly try to address social and structural determinants of health, including within their own practice, to reduce health inequities (Sowers et al., 2023). Recognizing this, in 2022, the Centers for Medicare and Medicaid Services (CMS)—the largest provider of public health insurance in the United States—expanded its strategic mission to include the advancement of health equity in supporting disadvantaged communities (CMS, 2022). One way of doing so is by requiring Medicare-managed care plans to conduct quality improvement (QI) projects that address health inequities or the provision of culturally and linguistically responsive care (Mutha et al., 2012).

QI initiatives aim to continuously evaluate and improve internal processes within specific clinics or health systems (Batalden & Davidoff, 2007). Utilization of the QI framework in medicine has been shown to improve health outcomes and reduce health disparities (Wells et al., 2004) (Sehgal, 2003). QI efforts can involve clinical care, as well as a non-clinical activities. Examples of each include conducting data-driven activities to improve patient safety, implementing and evaluating a new clinical program or administering medical education surveys and other system-based or administrative-related initiatives.

Practitioners in public and community psychiatry settings should have a solid understanding of QI as a framework for addressing structural issues, including by integrating a health equity lens in advancing these initiatives. There is some evidence that practitioners successfully apply QI frameworks to address systems-based issues across different clinical settings (Unutzer et al., 2002; Eckert et al, 2018; Kiger & Bertagnoli, 2018; Li et al., 2020). To further promote the use of QI among practitioners, the Accreditation Council for Graduate Medical Education (ACGME) competencies include a requirement for residents to demonstrate QI competency and “the ability to analyze the care they provide, understand their roles within health care teams, and play an active role in system improvement processes” (ACGME, 2023; Tomolo et al., 2009). Additionally, a core competency for psychiatry training involves equipping residents with awareness and understanding of system-based practices, including an ability to respond to “*the structural and social determinants of health and to call effectively on other resources to provide optimal health care*” (ACGME, 2023).

Integrating opportunities for trainees to engage in QI practices ensures they are equipped to embark on their future unsupervised practice with knowledge around these important frameworks and valuable skill sets to execute QI measures advancing systems of care. Given these required ACGME competencies and the framework outlined by agencies governing the delivery of care and access, it is important to increase the knowledge base and ability to execute QI initiatives for trainees and mental health providers at-large, towards advancing an equitable healthcare system (Reardon et al., 2020) (Ewins et al., 2017).

## Aims of the Current Report

We aim to demystify the QI process to make it more accessible to providers interested in making structural changes in their local clinical settings. Using examples from four projects conducted during the 2022–2023 academic year at the University of California, San Francisco (UCSF) Public Psychiatry Fellowship at Zuckerberg San Francisco General Hospital (ZSFG), we describe real-world implementation of QI efforts with an equity lens in community psychiatry settings, highlighting currently available best practices for conducting this work andstrategies for overcoming barriers throughout the process. Finally, we reflect on the sustainability of QI in community mental health settings.

## Context: Public Psychiatry Fellowship Capstone Project

Fellows in the UCSF Public Psychiatry Fellowship at ZSFG are placed in clinical settings in the San Francisco Bay Area serving publicly insured and uninsured individuals from marginalized communities for one year (Mangurian et al., 2014). Each fellow has both immersive clinical experience in a community setting, as well as an opportunity to develop and execute a QI-based capstone project within their clinics or the broader local behavioral health system. The overall objective of this element is to provide experience in leading QI initiatives to promote sustainable change in mental health services delivery for their clinical site or health system. The fellowship provides supporting resources, including a supervisor, research assistant, and opportunities for scholarship dissemination (Elser et al., 2020).

Within the first few months of the fellowship, fellows explore potential areas of needs within their clinic settings, in collaboration with their clinical site supervisor and the capstone project supervisor, with an equity-minded approach for their project. The aims and scope of the QI projects are informed by the fellows’ clinical and professional interests, their clinics’ needs and priorities, and the feasibility of being completed by the end of their fellowship year. All projects are supported by the capstone project supervisor who has expertise in health services research in public mental health settings, as well as a research assistant whose role includes conducting literature reviews to identify existing tools or practices, supporting the creation of any data collection instruments, and conducting data analysis. Fellows receive weekly supervision from the capstone project supervisor and feedback from all the fellowship faculty through four internal presentations throughout the year.

## Case Example: 2022–2023 QI Projects

**Fig. 1:**
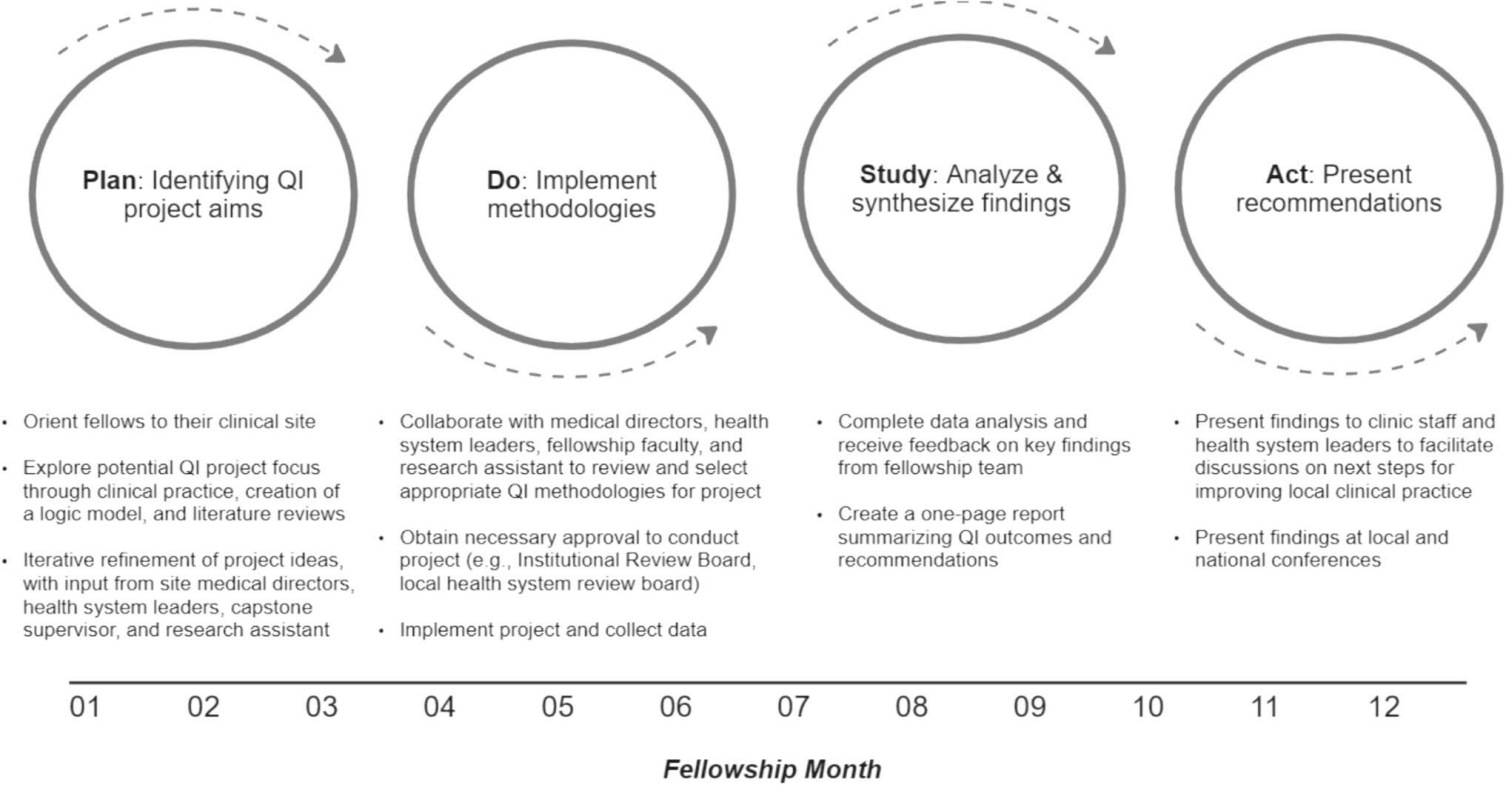
Quality improvement process

Three of the four fellows focused their QI projects on substance use disorder (SUD) assessment and treatment within public specialty mental health settings. Like many cities across the country, San Francisco has a high rate of unintentional fatal drug overdose, with Black/African Americans disproportionately impacted at 5 times the city-wide rate (Coffin et al., 2022). Many people seen in the fellows’ clinical sites have co-occurring mental health and substance use treatment needs and fellows observed barriers to accessing medication for SUD and other evidence-informed care for this population. For their QI projects, within each fellow’s respective clinical sites, they aimed to assess how providers were screening and assessing for SUDs, prescribing medication for SUDs, referring patients to relevant treatment resources, or engaging in shared decision-making (Karno et al., 2021; Marshall et al., 2022). Fellows received input on their project conceptualization, aims, methods, and relevant existing data, from the fellowship team and their clinical setting’s medical directors (Fig. 1). All fellows consistently met with the capstone project supervisor, medical directors, and key health system leaders to gain their input and reflections on their QI project designs and explore strategies to facilitate their clinic or health system’s engagement in the work (Fig. 1).

For the substance use-related projects, data collection methods included:

A) Administration of confidential online surveys to better understand screening practices at intake, treatment of SUDs, and perceived barriers and enabling factors to prescribing medication for alcohol, opioid, and nicotine use disorders. Several survey questions were adapted from existing published literature on barriers and facilitators to SUD treatment within similar clinical settings (Louie et al., 2019; Logan et al., 2021; Lanham et al., 2022). One fellow surveyed mental health providers in their clinic, and another surveyed prescribers within the broader behavioral health system.
B) Interviews with psychiatrists and case managers to obtain a more nuanced understanding of their perspectives around SUD treatment approaches such as shared-decision making, and potential challenges and best practices when treating individuals with SUD within community mental health settings.
C) Review of intake documentation in the electronic health record to better ascertain patterns around SUD diagnostic practices, psychoeducation about substance use, and resource referral patterns over a 6-month period.

The fourth fellow’s projects sought to better understand the current demographics of the prescriber workforce to help inform targeted recruitment and retention efforts and the provision of culturally congruent care for patients. This project aim was identified by the health system as a major priority and was bolstered by recent research illustrating the importance of workforce diversity in addressing health inequities facing minoritized communities. Most notably, there is some evidence that racial concordance between providers and patients can lead to improved health outcomes, lower mortality rates, and improved satisfaction with care (Hill et al., 2018, 2023; Snyder et al., 2023). Thus, to address health inequities facing racialized groups, the fellow’s project aimed to address the urgent need to advance workforce diversity as part of the strategic solution. A confidential online survey was administered to behavioral health prescribers within the entire health system to understand their demographics (e.g., race, ethnicity, gender identity, sexual orientation, language proficiency, first-generation status), perspectives on factors impacting their own recruitment and retention, and experiences with diversity and inclusion within the workplace. The survey creation involved expert consultation and review of research on current evidence-informed/best practices for curating an equity-minded demographic survey (Flanagin et al., 2021; The White House, 2022; OMB, 2023). Questions about recruitment and retention were drawn from a validated questionnaire examining diversity and inclusion within academic medicine (Person et al., 2015).

At the end of the academic year, all fellows presented a one-page report of their QI project findings and initial ideas about their clinical implications to their clinic and health system leaders. During these presentations, medical directors and front-line providers were able to ask questions and provide input regarding the project findings, with the aim of considering potential next steps for the work and improving local practice. The current manuscript's aim is not to describe in-depth the methodology and results of each project but to discuss the process of executing the work, including challenges that arose and strategies for overcoming them. For reference, a summary of all the QI projects’ primary aims, measures, and proposed changes based on their findings has been outlined below (Table 1) using the Institute of Healthcare Improvement’s QI framework (Langley et al., 2009).

**Table 1:** 2022–2023 Public psychiatry QI project outlines

Aims	Measures	Proposed changes
Expanding data collection on patient and provider demographics	Race/ethnicity Languages spoken Gender identity Sexual Orientation	Conducting systemwide disaggregation of both patient and provider ethno-racial data to better understand the need for culturally congruent care Creating an accessible and user-friendly system for gathering and analyzing data
Addressing social determinants of health and barriers to SUD treatment access	Housing security Social isolation Trauma Referral wait times	Aligning QI work with on-going clinic/system initiatives and goals Partnering with academic and community agencies to advance initiatives and expand organizational and patient-specific resources
Implementing evidence-informed treatment practices for SUD in public mental health community clinics	Screening rates Assessment rates Prescribing rates Familiarity with SUD resources Provider confidence levels in prescribing medication for SUD	Establishing ongoing conversations with clinic leaders, staff, and prescribers to identify the most salient areas for SUD-related initiatives Providing expert consultation and guidance on how to prescribe SUD medication for those with complex psychosocial needs Supporting patient empowerment, motivational interviewing, and shared decision-making when feasible
Improving workforce diversity, retention and staff support	Provider caseload Workplace diversity and inclusivity initiatives Workplace satisfaction Patient-provider concordance (Race/ethnicity, languages spoken, gender, sexual orientation) First-generation student and health professional status	Increasing access to professional development opportunities and advancing organizational cultural competency Recognizing context of how QI may add to the existing workload of overburdened workforce; getting leaders’ buy-in who can create protected time for folks to participate

## QI Challenges, Strategies, and Sustainability Considerations

Multiple challenges arose during each of the fellows’ projects. We highlight some of the more common challenges faced in QI work and offer lessons learned for overcoming them to promote sustainable change.

## Transparency Factors

In all projects, regular communication with clinical and administrative staff, as well as health system leaders was crucial. Prior to beginning their projects, all fellows conducted logic models of their clinics to better understand the organizational landscape. This was beneficial in promoting understanding of key players within the complex systems in which they were working. In addition to ensuring buy-in from clinic leadership (e.g., medical directors), fellows also presented their proposed initiatives to others in the clinic (e.g., front-line clinicians) to ensure the relevance of the work and foster engagement and trust. The specific method of communication differed, for example presenting in clinical team meetings about their QI work to solicit participation, sending regular email updates to leadership about progress, and speaking more informally with colleagues in the clinic to engage them in the work.

For certain aspects of fellows’ data collection (e.g., demographic information), some systems partners were concerned about how the collected protected information would be used. To address these concerns, fellows maintained ongoing correspondence and engagements focused on optimizing transparency around why and how information was being collected, including the handling of data confidentiality. For example, the clinical leadership in the system did not have direct access to individual data but rather received aggregate reporting of demographic factors from the fellow and UCSF team. It was also important to acknowledge that despite best efforts, not everyone in the system may trust the process outlined and some may choose not to participate, and this could be based on identity-related factors, part of a larger societal context, and/or mistrust of the targeted systems-at-large.

## Resource and Time Constraints

Since fellows were expected to complete their QI work within a year, there was an expedient need to understand the potential bureaucratic hurdles to advancing the work within their respective health systems quickly. Timely consideration of any administrative delays and engagement with appropriate stakeholders in each specific system is of utmost importance for assessing the overall timeline of QI work. This included getting early buy-in from leadership who can advocate at higher levels if larger administrative delays arise. For example, the fellows were required to get an exemption from the UCSF Institutional Review Board and the local public health systems’ research review boards. These review processes can be lengthy and having regular communication with both system leadership and the review boards was vital to successful completion of the works within the fellowship year. Additionally, integrating a needs assessment and program evaluation analysis early in the formation of the QI process can ensure the work being done is of direct benefit to the system and can help the redundancy of efforts.

Finally, there needs to be a careful consideration of methodology or goals pursued based on the available time and resources—this may necessitate exploring different revenue sources and external partnerships. Collaborating with others can help optimize sustainability, enhance efficiency, amplify the work, and limit the risk of overburdening the workforce. This can also help mitigate the concerns around “wasted efforts,” a common concern around QI’s ultimate meaningfulness and impact (Dixon-Woods & Martin, 2016). For example, having protected time to execute the project amidst other ongoing clinical responsibilities requires leadership prioritization of these initiatives and their integration into the organization's overall goals and missions. There could also be additional considerations around avenues of rewarding and incentivizing QI work including as part of career advancement strategies. For projects focused on equity-specific goals, there should be an intentional focus on minimizing the perpetuation of the “minority tax” which can disproportionately impact minoritized individuals who are most likely to advance these equity-based initiatives (Campbell & Rodriguez, 2019; Jordan et al., 2021). Requiring leadership roles to advance health equity may be an incentive to facilitate their support and longitudinal engagement in these initiatives (Jordan et al., 2021).

## Unexpected Directions

When planning any QI work, it is integral to be flexible and adaptable based on evolving context and gained insight, including an enhanced understanding of system needs, feedback received, and potential barriers that arise. As the work progresses, there is often a need to modify initial objectives and plans; data-driven adaptations are a natural part of the QI process and do not necessarily indicate a failure or need to terminate prematurely. For example, one of the fellows initially aimed to conduct both interviews and a survey to examine barriers to prescribing substance use medications. However, given concerns about time constraints, the fellow discontinued the interviews and focused on maximizing the impact of the survey findings among prescribers and behavioral health system leaders. It is essential to recognize that with QI processes, particularly when conducted by learners who have time-limited placements, one project—no matter how seemingly small—can lay a crucial foundation for subsequent projects and efforts that can ultimately yield systemic impact.

## Lack of Infrastructure for QI

It is important to acknowledge that the resources provided by the fellowship–such as a research assistant, supervisor with expertise, data analyst, and dedicated time–may not be available to all community psychiatry providers. Nonetheless, pre-existing resources through agencies such as CMS, can be utilized to provide support for QI initiatives for clinics serving Medicaid and Medicare populations.

It would be beneficial for community psychiatrists to consider multisector local and regional partnerships to leverage other work groups within the broader health system and university research resources. Additionally, given the burgeoning of technological advancements, including artificial intelligence—a rapidly evolving space for healthcare in general—exploring the integration of these tools could potentially help advance QI initiatives (Feng et al., 2022).

## Conclusion

QI initiatives are an important tool in advancing healthcare needs across different settings. By utilizing examples of QI initiatives driven by previous fellows as a guiding framework, we provide an overview of best practices for the design, methodology, and structure of QI work that could be scaled in other clinical training program settings. Our paper also explores the challenges and opportunities of QI implementation in community mental health settings through an equity lens, including the consideration of transparency factors, resource and time constraints, unexpected directions, and lack of infrastructure for QI. By reflecting on strategies for optimizing partnerships between academic and public sector organizations, we hope our findings help make QI more accessible for providers trying to make structural changes within their local clinical settings.
